# Quantification of Polyethylene Glycol 400 Excreted in the Urine by MALDI-TOF Mass Spectrometry

**DOI:** 10.3390/pharmaceutics14071341

**Published:** 2022-06-24

**Authors:** Ákos Kuki, Mahir Hashimov, Tibor Nagy, Csaba Tóth, Miklos Zsuga, Sándor Kéki

**Affiliations:** 1 Department of Applied Chemistry, Faculty of Science and Technology, University of Debrecen, Egyetem tér 1, H-4032 Debrecen, Hungary; kuki.akos@science.unideb.hu (Á.K.); mahir.hashimov@science.unideb.hu (M.H.); nagy.tibor@science.unideb.hu (T.N.); zsuga.miklos@science.unideb.hu (M.Z.); 2 Doctoral School of Chemistry, University of Debrecen, Egyetem tér 1, H-4032 Debrecen, Hungary; 3 Today’s Life Science and Research Kft., Bulcsú utca 20/A, H-2120 Dunakeszi, Hungary; tothcsaba.pk30@gmail.com

**Keywords:** polyethylene glycol, intestinal permeability, MALDI-TOF, quantification

## Abstract

Polyethylene glycol 400 (PEG 400) was used as a permeability probe to examine the gastrointestinal tract which can be involved in the pathogenesis of some inflammatory and autoimmune diseases. A novel methodology was developed and validated for the quantitation of PEG 400 excreted in human urine after oral administration using matrix-assisted laser desorption/ionization mass spectrometry (MALDI-MS). The excretion ratios were determined for the most intense ions corresponding to nine PEG 400 oligomers. The relative error of accuracy was between –6.0% and 8.5%, and the relative standard deviation (RSD) of the precision was below 15%. Our method was successfully applied in a large-scale experimental study involving nearly two hundred volunteers. Due to the large number of measurements, detailed and reliable statistical analysis was performed. No significant difference was found between the male and female group of volunteers at 0.05 significance level, except the two largest PEG oligomers. However, the average excretion ratios of the male volunteers are greater than that of the women for all the nine PEG oligomers, suggesting a difference in the intestinal permeability between men and women.

## 1. Introduction

Polyethylene glycol (PEG) is a linear and chemically inert compound that can be produced in various chain lengths and molecular weights [1,2]. PEG 400, polyethylene glycol with an average molecular weight of 400 g/mol, has low toxicity and is commonly used as an inactive ingredient in the pharmaceutical industry as a solvent, plasticizer, surfactant, ointment and suppository base, drug delivery candidate, tablet, and capsule lubricant [3,4,5,6,7,8]. Another most important advantage of PEG 400 in medicine is that it is an ideal permeability probe for studying the gastrointestinal tract because of its high hydrophilic or lipophobic properties, inert nature, absence of metabolism by intestinal bacteria after absorption, and rapid excretion in urine [9,10,11,12,13]. An altered small intestinal permeability may be implicated in disease, ulcerative colitis, celiac disease, rheumatoid arthritis, and many allergic diseases [14]. Clinical trials have also found an association with cardiovascular disease [15]. Intestinal permeability tests are quite sensitive for the detection of these kinds of diseases [16,17,18,19,20]. It is usually investigated by ingesting of 2–10 g of PEG 400 after an overnight in a fixed volume of water, and urine specimens are collected for 5–6 h for analysis.

There are several methods to analyze and quantify PEG in urine such as high-performance liquid chromatography (HPLC) [21], gas–liquid-chromatography (GLC) [22,23], gel permeation chromatography (GPC), gas-chromatography–mass spectrometry (GC–MS) or liquid-chromatography–mass spectrometry (LC–MS) [24], and nuclear magnetic resonance (NMR) spectroscopy [25]. These methods are time and labor-consuming, requiring longer processing time and/or extensive sample pre-treatment such as centrifugation, derivatization, extraction, etc. Some of these methods, however, are of lower sensitivity and only applicable to analyze higher doses of PEG 400. Direct injection electrospray ionization mass spectrometry (ESI-MS) can significantly increase the analytical throughput compared to the hyphenated techniques (e.g., LC–MS) [26]. However, matrix-assisted laser desorption/ionization (MALDI)—the other main soft ionization technique—can offer some advantages over ESI. For example, MALDI is less susceptible to ion suppression from salts and allows a large number of samples to be introduced into the ion source simultaneously, having thereby a higher throughput potential than ESI. Furthermore, quantification with MALDI-MS can be achieved by mixing standard compounds in analyte mixtures. Furthermore, internal standards with similar chemical structures to that of the analyte minimize the uncertainties in quantitative analysis [27].

In this work, we report a novel method for the quantification of PEG 400 excreted in urine by MALDI coupled to time-of-flight (TOF) mass spectrometry. In addition, we determined not only the overall intestinal permeability of PEG 400, but that of each individual prominent oligomer of PEG 400.

## 2. Experimental

### 2.1. Chemicals

PEG 400 for the intestinal gut permeability tests were purchased from a pharmacy as macrogol 400, while the methoxy polyethylene glycol (mPEG) was purchased from Sigma Aldrich (Steinheim, Germany). Methanol (VWR International) and 18.2 MΩ cm (MilliQ grade) water were used as solvents.

### 2.2. Collection and Preparation of Urine Samples

Urine samples were collected at pre-dose and after the administration of 3 g PEG 400 in a 6 h interval and provided by International Center for Medical Nutritional Intervention (ICMNI) Orvosi Rehabilitacios Centrum Ltd (Szigliget, Hungary). Urine samples were diluted with water to extend the volume to approximately 750 mL where it was required. A volume of 1 mL was taken from this solution and spiked with 50 μL mPEG (c: 15 mg/mL of mPEG 750) serving as internal standard (IS), and diluted two-fold prior the MALDI analysis in order to reduce the salt concentration. The excretion ratios of the PEG 400 oligomers (PEG*_n_*, number of repeat units *n* = 6–14) were calculated as follows: *excretion ratio* (%) = 100 × *(PEG_n_ concentration in the sample* × *volume of urine* (mL)*)/amount of PEG_n_ administrated*. The weight ratios of PEG oligomers in PEG 400 were calculated based on the molecular weight distribution of pure PEG 400 as determined by MALDI-TOF.

### 2.3. Preparation of Standard and Quality Control Solutions

Pooled human urine was collected at pre-dose; 9 calibration solutions were created with the approximate concentration of 0.075, 0.225, 0.450, 0.600, 0.750, 0.900, 1.050, 1.200, and 1.350 mg/mL (PEG 400); 1 mL of these standard solutions were spiked by 50 µL of internal standard and diluted two-fold prior to MALDI-TOF analysis. The standard std3 (c: 0.450 mg/mL) and std8 (c: 1.200 mg/mL) were selected as lower and higher concentration quality control samples (LQC, HQC).

### 2.4. Matrix-Assisted Laser Desorption/Ionization Time-of-Flight Mass Spectrometry (MALDI-TOF-MS)

The measurements were carried out by an Autoflex. Speed MALDI-TOF-MS instrument (Bruker Daltonik, Bremen, Germany). Reflectron mode was used where the ion source voltage 1, ion source voltage 2, reflector voltage 1, and reflector voltage 2 were 19 kV, 16.65 kV, 21 kV, and 9.55 kV, respectively. The instrument is equipped with a solid phase laser (355 nm).

The spectra were calibrated by PEG 400 externally. The MALDI matrices were dissolved in methanol in a concentration of 20 mg/mL. The ionizing agent was sodium trifluoroacetate (5 mg/mL). The matrix, the ionizing agent, and samples were mixed in the volume ratio of 20:2:5, respectively; 0.5 μL of the solution was deposited onto a metal sample plate and allowed to air-dry. The spectra were evaluated by the FlexAnalysis 3.4 software (Bruker).

### 2.5. Method Validation

The quantification of PEG*_n_* (*n* = 6–14) oligomers was based on the internal standard (IS) method. The calibration curves were constructed using the peak height ratios of an oligomer ion to the sum of five prominent ions of the IS series. The standard solutions were spotted ten times on the MALDI target plate resulting in ten data points for each concentration. The precision and accuracy were determined based on ten replicates at two concentration levels: LQC (0.450 mg/mL of PEG 400) and HQC (1.200 mg/mL of PEG 400) spiked into pre-dose urine. Precision was expressed as a relative standard deviation (RSD%), while the accuracy (%) was calculated as *(average calculated concentration)/(nominal concentration)* × 100. The limit of detection (LOD) and limit of quantification (LOQ) are the concentrations at which the MALDI-MS signal level of the PEG*_n_* (*n* = 6–14) oligomers reaches at least 3 and 10 times the signal noise of the baseline, respectively. Each day, the analytical sequence consisted of running 9 calibration solutions (10 replicates for each), followed by the analyses of the post-dose urine samples (3 replicates for each). Additional details about the method validation can be found in the Section 3.

### 2.6. Data Analysis

The MALDI-MS spectra were evaluated with the Compass flexAnalysis 3.4 software (Bruker Daltonics, Bremen, Germany). The statistical analyses were performed by the Microsoft Excel Analysis ToolPak (Microsoft Office Professional Plus 2016, version:16.0.4266.1001, Microsoft, Redmond, WA, USA).

## 3. Results and Discussion

### 3.1. Method Development

Initially, PEG 400 standard was analyzed using 2,5-dihydroxybenzoic acid (DHB) as the MALDI matrix and Na^+^ as the ionization agent.

**Figure 1:**
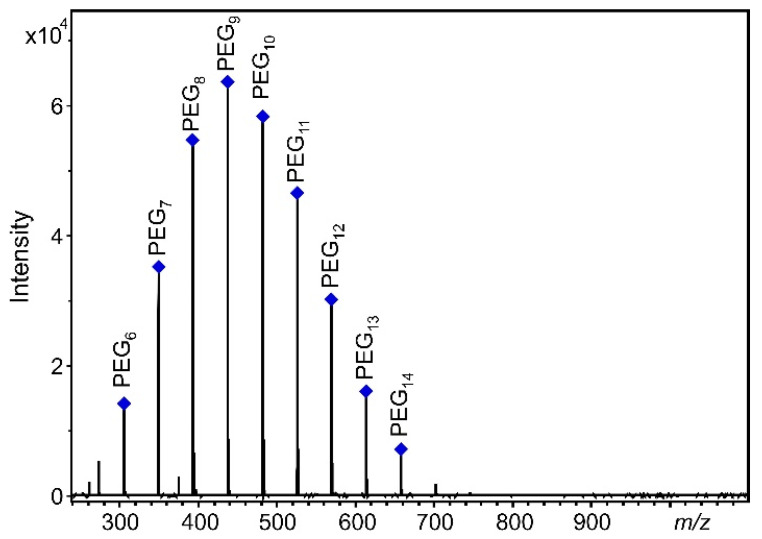
MALDI-TOF-MS spectrum of PEG 400 with DHB matrix and Na^+^ ionization agent.

As seen in Figure 1, with the addition of Na^+^, a single series of PEG 400 ions completely dominate the spectra. The [H(C_2_H_4_O)_n_OH + Na]^+^ ions at *m*/*z* 305, 349, 393, 437, 481, 525, 569, 613, and 657 correspond to PEG 400 oligomers with the number or repeat units *n* = 6–14 (PEG_6_–PEG_14_). It demonstrates one of the main advantage of MALDI-TOF-MS, namely that the adduct ion formation can effectively be altered by the addition of appropriate ionization agent, and the minor series (e.g., the [H(C_2_H_4_O)_n_OH + K]^+^ ions) can also be suppressed. Moreover, in line with our goals, the appearance of only one PEG ion series facilitates the quantitative analysis.

**Figure 2:**
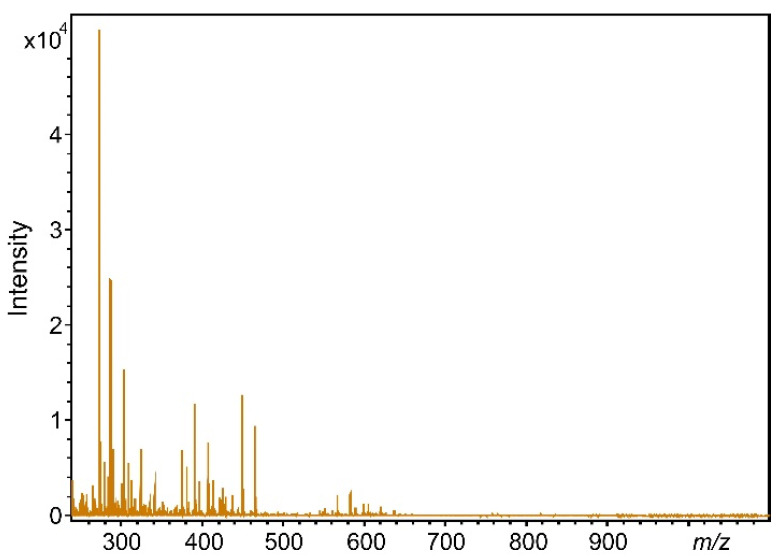
MALDI-TOF mass spectrum of a pre-dose human urine sample.

In the next step, pre-dose human urine was analyzed by MALDI-TOF-MS (see Figure 2). Urine is a highly complex biological fluid composed of organic and inorganic substances, for example, waste products of cellular metabolism and constituents derived from foods that are eaten. Therefore, sample preparation is crucial prior to introduction into the ion source of the mass spectrometer, especially when electrospray ionization (ESI) is used. However, a huge benefit of MALDI-TOF-MS, in our case, is that the sample preparation is simplified to dilution and matrix/ionization agent addition, and no further special extraction or filtration steps are required, even if the urine samples contain solid particles, e.g., precipitates. Figure 2 demonstrates that the mass spectra of the urine samples show patterns of wide variety, influenced by, e.g., the foods which have been eaten, as mentioned above.

**Figure 3:**
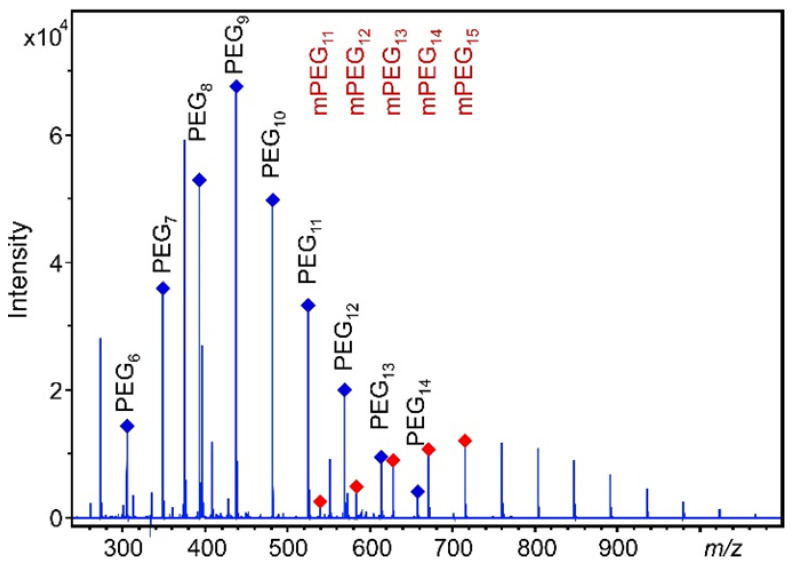
MALDI-TOF spectrum of a pre-dose human urine sample spiked with PEG 400 at 0.900 mg/mL and 50 µL of mPEG. The peaks marked by red symbols denote the mPEG oligomer series.

Figure 3 shows the MALDI-TOF-MS spectrum of a pre-dose human urine sample spiked with PEG 400 at 0.900 mg/mL and 50 µL of methoxy polyethylene glycol (mPEG) as an internal standard (IS). (The internal standard-based calibration using mPEG will be detailed later). As seen in Figure 3, no interference of mass spectral peaks was observed from the urine constituents or MALDI matrix that makes quantification of PEG oligomers feasible.

**Figure 4:**
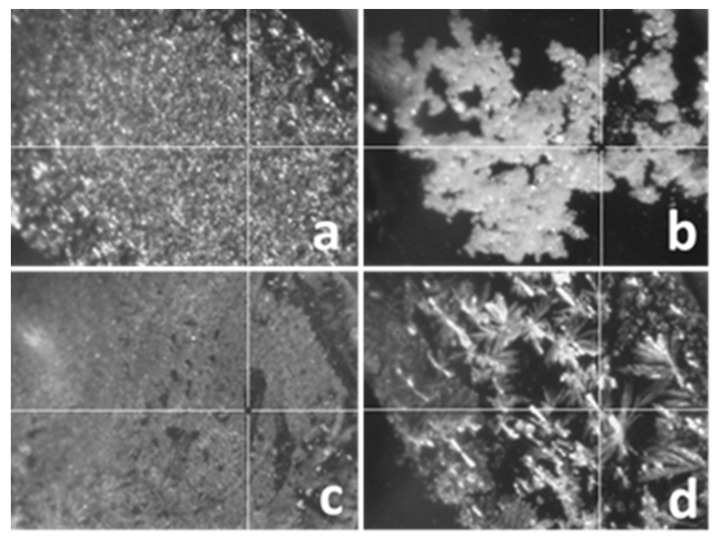
MALDI camera images of the pre-dose human urine samples spiked with PEG 400 deposited on the MALDI target plate using various matrices: (**a**) DHB; (**b**) DCTB; (**c**) Dithranol; (**d**) THAP (magnification: 50).

During sample preparation for MALDI-TOF-MS, a suitable matrix compound is mixed with the analyte and deposited onto a ground steel target plate. The quality and reproducibility of the MALDI-TOF mass spectra strongly depends on the choice of the MALDI matrix. For quantification, homogenous deposition resulting in good reproducibility is more important than a supreme sensitivity or signal-to-noise ratio [28,29]. To choose the best matrix suitable for the analysis of human urine samples spiked with PEG 400, we tested four different matrix compounds including 2,5-dihydroxybenzoic acid (DHB), trans-2-[3-(4-tert-butylphenyl)-2-methyl-2-propenylidene]malononitrile (DCTB), 1,8,9-anthracenetriol (dithranol), and 2,4,6-trihydroxyacetophenone (THAP). Figure 4 illustrates the homogeneity and crystallinity of the deposited analyte-matrix mixture.

As seen in Figure 4, DHB shows more homogeneity than all of the others. It is in line with the reproducibility of the repeated measurements, i.e., the intensity variation of the MALDI-TOF-MS peaks of the PEG series is the lowest with DHB. As none of the matrices tested displayed superior resolution or signal-to-noise ratio, DHB was chosen for the next experiments.

**Figure 5:**
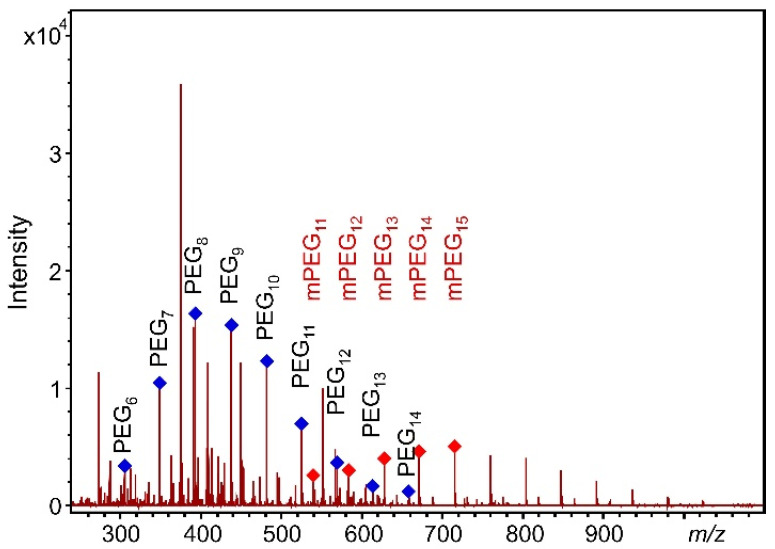
MALDI-TOF mass spectrum of a urine sample from a volunteer following administration of PEG 400 spiked with 50 µL of mPEG as internal standard. The peaks marked by red symbols denote the mPEG oligomer series.

Absolute quantification was performed by adding a fixed amount of mPEG with an average molecular weight of approximately 750 Da to the samples as the internal standard. Figure 5 reveals a representative MALDI-TOF mass spectrum of a urine sample from a volunteer following administration of PEG 400 spiked with mPEG. In order to construct calibration curves, pre-dose human urine samples spiked with fixed amount of the internal standard and varying amounts of pure PEG 400 were measured (see Figure 3). Separate calibration curves were constructed for each PEG 400 oligomer (PEG_6_–PEG_14_) using the peak height ratios of an oligomer ion to the sum of five prominent ions of the mPEG series. Thus, the ion intensity ratios were converted to absolute amounts.

### 3.2. Method Validation

The validation was performed according to the International Conference on Harmonization Guideline for the Validation of Analytical Procedures [30]. It was based on the following criteria: limit of detection (LOD), limit of quantification (LOQ), precision, accuracy, and linearity. The validation data are reported in Table 1.

**Figure 6:**
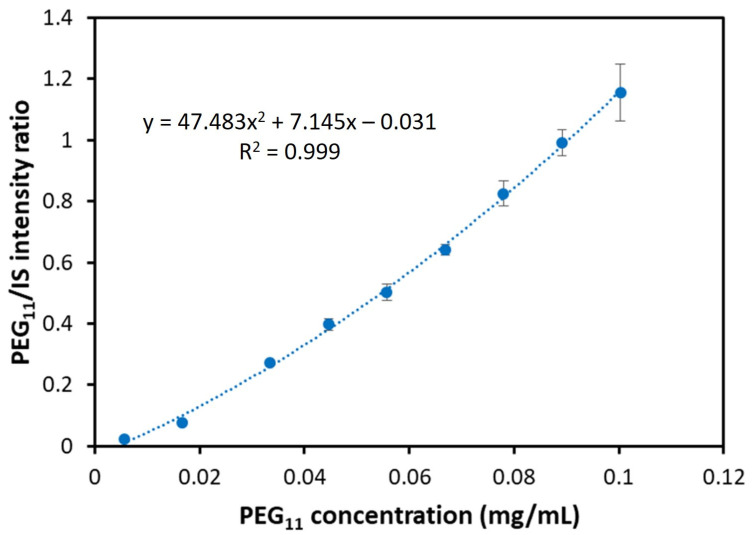
Calibration curve for PEG_11_. The PEG_11_/IS ion intensity ratio is plotted against the PEG_11_ concentration. Error bars represent the standard deviation.

Figure 6 shows a representative calibration curve for the PEG_11_ oligomer. As seen in Figure 6, the visual inspection of linearity suggests a polynomial regression model. Therefore, the linearity of the calibration process was investigated by means of the Lack-of-fit (LOF) test [31]. The LOF results indicate that the linear regression model is inadequate, and the linearity of the calibration lines was rejected at the 95% confidence level: F = 15.36 (F_crit,95%_ = 2.125) and *p* = 0.00000. Alternatively, the U-shape residual plot of the linear regression (see Supplementary Figure S1) also indicates that a quadratic regression model (QRM) should be preferred over the linear one. The test for LOF reveals that QRM adequately fits the calibration data at 95% confidence level: F = 2.080 (F_crit,95%_ = 2.213) and *p* = 0.065. Furthermore, the significance of the second order regression coefficient is confirmed (*p* = 0.0005), the residuals of the QRM were randomly scattered (see Supplementary Figure S2), and the y-intercept of the QRM curve is not significantly different from zero (*p* = 0.128). (The corresponding p value of the linear regression model is *p* = 0.008 which indicates a significant y-intercept). The adjusted R square (R^2^_adj_ = 0.9980) is roughly the same as the R square (R^2^ = 0.9985) meaning that the QRM is quite robust. (It must be noted that the adjusted R^2^ value of the QRM is greater than that of both the linear and 3rd order polynomial regression model). Furthermore, as seen in Table 1, the R square values for the other PEG oligomers also support the application of QRM (R^2^ from 0.996 to 0.999).

The statistical data summarized in Table 1 reveal good accuracy and precision: the relative error of accuracy was between –6.0% and 8.5%, and the relative standard deviation (RSD) of the precision was below 15% for all the lower concentration and higher concentration quality control samples (LQC, HQC).

### 3.3. Urinary Excretion Results

**Figure 7:**
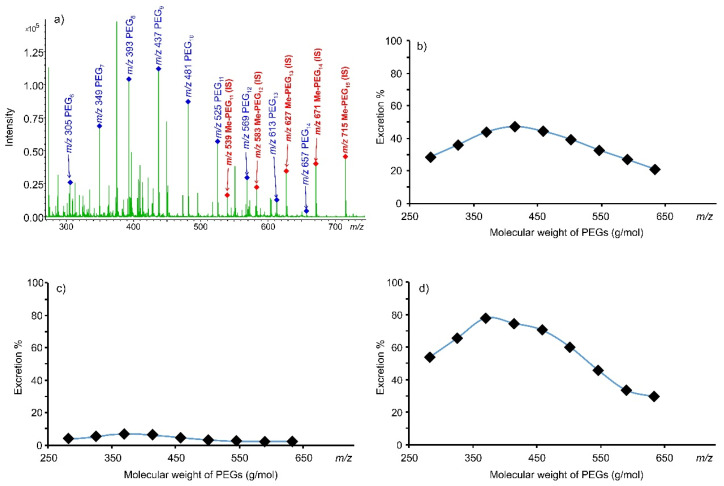
(**a**) MALDI-TOF mass spectrum of a urine sample from a volunteer following administration of PEG 400 spiked with 50 µL of mPEG as internal standard; (**b**) Excretion of the PEG_6-14_ oligomers calculated by our method based on the spectrum shown to the left; (**c**,**d**) Extreme excretion examples.

We applied our method in a large-scale experimental study involving 197 volunteers. Figure 7a,b show the MALDI-TOF mass spectra of the urine sample from a volunteer after the administration of PEG 400, and the corresponding excretion ratios of the PEG_6–14_ oligomers determined by our method. Two additional extreme excretion ratio plots are given in Figure 7c,d, expressing the remarkable differences in the intestinal permeability among the volunteer subjects.

**Figure 8:**
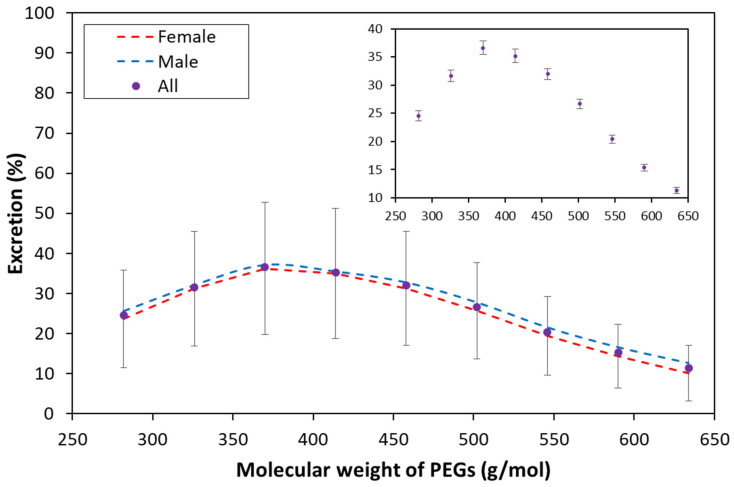
The average excretion ratios of the PEG_6–14_ oligomers based on a sample of about 200 volunteers. The error bars represent the standard deviation of the subjects and the standard error of the mean in the main plot and in the inset, respectively. The red and blue regression curves belong to the average excretion ratios of the female and male volunteers, respectively.

The size of the sample enables us to make a reliable statistical analysis about the intestinal permeability of the PEG 400 oligomers. The average excretion ratios are plotted in Figure 8, and the descriptive statistics are given in Table 2.

The large standard deviation values indicate that the excretion ratios of the subjects can spread far from the mean, as the two extreme examples in Figure 7c,d demonstrate. However, the excretion ratio mean values have very small standard errors (see Figure 8 inset), supporting that accurate estimates are given for the corresponding population parameters. The analysis of variance (ANOVA) test shows that the size of the PEG oligomers has a significant effect on the excretion ratios (*F* = 93, *F_crit_* = 1.94, *p* = 0.0000). However, there is no significant difference between the permeability of the PEG_8_ and PEG_9_ oligomers (*p* = 0.38). The two sample T-tests for all the other adjacent PEG oligomer pairs show significant differences between their permeabilities (*p* < 0.036).

It is very interesting to investigate whether there is a difference in the intestinal permeability between men and women. Both Figure 8 and Table 2 show the excretion data and statistics by grouping them into male and female. In Table 2, the descriptive statistics are supplemented by the *p* values of the two-sample (male/female) *t*-tests. The tests clearly demonstrate that there is no significant difference between the male and female group at *p* < 0.05 significance level, except the two largest PEG oligomers. It must be noted, however, that if the significance threshold is set at a stricter level *p* < 0.01, then the male/female differences in the permeability of PEG13 and PEG14 are also insignificant. Moreover, the average excretion ratios of the male volunteers are higher for all the PEG oligomers than those of the women (as the regression lines indicate in Figure 8), suggesting a difference in the intestinal permeability between men and women.

## 4. Conclusions

A novel method was developed and validated for the quantification of PEG 400 oligomers excreted in human urine by MALDI-TOF mass spectrometry. Our methodology was successfully applied for the urine samples of about two hundred volunteers (evenly divided between men and women). In this large-scale experimental project, we had the opportunity to experience the advantages of MALDI-TOF-MS analysis of these complex samples, namely the simplified sample pre-treatment, the high-throughput, and the ease of data processing. Furthermore, the large sample size allowed to gain statistically valid results. Although the excretion ratios of the individual subjects spread far from the average, the mean values were determined with very small standard errors, offering thereby accurate estimates for the corresponding population parameters.

## Figures and Tables

**Table 1:** The R^2^ values of the quadratic regression model, accuracy, precision, limit of detection, and limit of quantification of the PEG oligomers in human urine. The mean values of *n* replicates are presented.

PEG	Calibration Curve Equation	R^2^ *n* = 10	Adjusted R^2^ *n* = 10	Accuracy (%) *n* = 10 LQC HQC		Precision (RSD %) *n* = 10 LQC HQC		LOD *n* = 5 (μg/mL)	LOQ *n* = 5 (μg/mL)
PEG_6_	319x^2^ + 8.72x − 0.002	0.998	0.997	99.5	95.8	12.1	5.6	0.48	1.60
PEG_7_	92.3x^2^ + 10.9x − 0.022	0.998	0.997	98.0	101.8	6.4	5.1	0.51	1.70
PEG_8_	47.2x^2^ + 10.9x − 0.016	0.997	0.996	101.7	106.9	7.8	3.8	0.45	1.50
PEG_9_	15.5x^2^ + 12.7x − 0.052	0.996	0.995	94.0	108.5	11.1	3.6	0.40	1.35
PEG_10_	30.8x^2^ + 9.45x − 0.044	0.998	0.997	100.0	107.8	6.0	2.8	0.58	1.92
PEG_11_	47.5x^2^ + 7.15x − 0.031	0.999	0.998	98.7	105.7	2.0	3.1	0.74	2.46
PEG_12_	76.2x^2^ + 5.38x − 0.015	0.999	0.998	94.8	103.0	2.1	2.3	0.78	2.58
PEG_13_	134x^2^ + 3.58x − 0.004	0.999	0.999	96.3	102.5	3.4	1.7	0.90	3.00
PEG_14_	234x^2^ + 2.69x + 0.000	0.999	0.999	96.6	105.1	6.5	2.4	0.71	2.63

**Table 2:** Descriptive statistics of the excretion ratios (%) of the PEG_6-14_ oligomers.

		PEG_6_	PEG_7_	PEG_8_	PEG_9_	PEG_10_	PEG_11_	PEG_12_	PEG_13_	PEG_14_
Mean	*Female*	23.6	31.2	36.3	35.0	31.3	25.7	19.4	14.3	10.2
	*Male*	25.6	32.0	37.1	35.5	32.7	27.8	21.5	16.6	12.6
	*All*	24.5	31.6	36.6	35.2	32.0	26.7	20.4	15.4	11.3
Standard Error		1.18	1.45	1.66	1.64	1.35	1.11	0.90	0.76	0.67
		1.27	1.45	1.66	1.63	1.52	1.31	1.06	0.85	0.77
		0.87	1.02	1.18	1.16	1.01	0.85	0.70	0.57	0.51
Standard Deviation		12.0	14.7	16.9	16.7	13.7	11.3	9.2	7.7	6.6
		12.3	14.1	16.1	15.8	14.7	12.7	10.3	8.2	7.2
		12.1	14.4	16.5	16.2	14.2	12.0	9.8	8.0	7.0
Minimum		6.2	9.3	9.5	7.8	7.2	5.8	3.9	1.3	0.0
		3.7	5.1	6.8	6.0	4.4	3.1	2.2	1.9	0.0
		3.7	5.1	6.8	6.0	4.4	3.1	2.2	1.3	0.0
Maximum		65.1	90.5	99.5	93.8	62.5	55.2	44.2	33.9	31.5
		56.7	66.3	77.8	74.6	70.5	60.1	45.9	34.5	33.0
		65.1	90.5	99.5	93.8	70.5	60.1	45.9	34.5	33.0
Count		103	103	103	103	103	103	103	103	97
		93	94	94	94	94	94	94	93	86
		196	197	197	197	197	197	197	196	183
Confidence Interval (95%)		2.35	2.87	3.30	3.26	2.69	2.20	1.79	1.50	1.33
		2.53	2.88	3.31	3.24	3.02	2.61	2.11	1.68	1.54
		1.71	2.02	2.32	2.28	2.00	1.69	1.37	1.12	1.01
*T*-test *p* values (female, male)		0.27	0.69	0.73	0.84	0.48	0.24	0.16	0.048	0.019

## Data Availability

Data is contained within the article or Supplementary Material.

## Supplementary Materials

The following supporting information can be downloaded at: https://www.mdpi.com/article/10.3390/pharmaceutics14071341/s1, Figure S1: Residual plot of the linear regression model of the calibration data for PEG_11_, Figure S2: Residual plot of the quadratic regression model of the calibration data for PEG_11_.
