# Supplementary material for: Quantification of Polyethylene Glycol 400 Excreted in the Urine by MALDI-TOF Mass Spectrometry
Source: Pharmaceutics. 2022 Jun 24;14(7):1341. doi: 10.3390/pharmaceutics14071341 (PMC9322888; doi:10.3390/pharmaceutics14071341)
Supplement: Supplementary file 1 [file pharmaceutics-14-01341-s001.zip › pharmaceutics-1696269-supplementary.pdf]

Supporting information

Quantification of polyethylene glycol 400 excreted in the urine by MALDI-TOF mass spectrometry

Ákos Kuki[1], Mahir Hashimov[1,2], Tibor Nagy[1], Csaba Tóth[3], Miklós Zsuga[1], Sándor Kéki[1,*]

[1]*Department of Applied Chemistry, Faculty of Science and Technology, University of Debrecen, Egyetem tér 1, H-4032 Debrecen, Hungary*

[2]*Doctoral School of Chemistry, University of Debrecen, Egyetem tér 1, H-4032 Debrecen, Hungary*

[3]*Today's Life Science and Research Kft., Bulcsú utca 20/A, H-2120 Dunakeszi, Hungary*

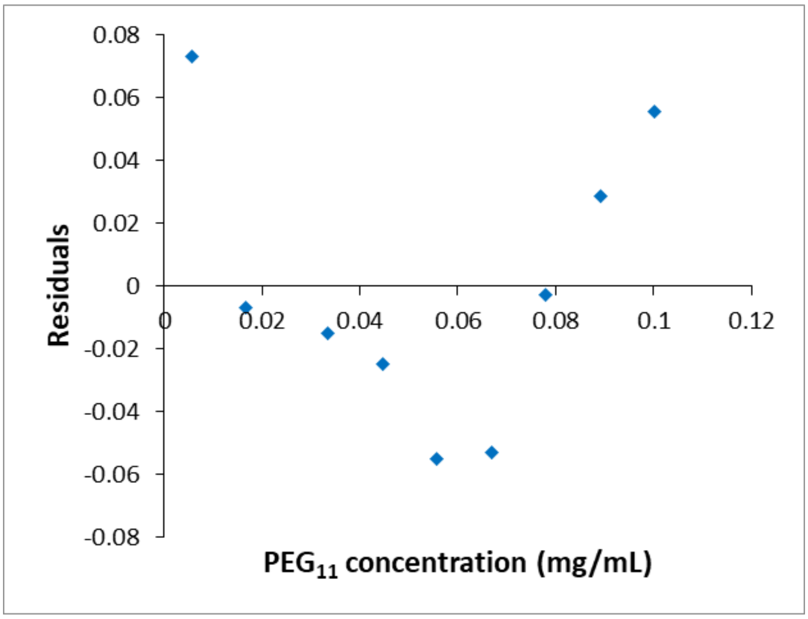

Figure S1. Residual plot of the linear regression model of the calibration data for PEG_{11}.

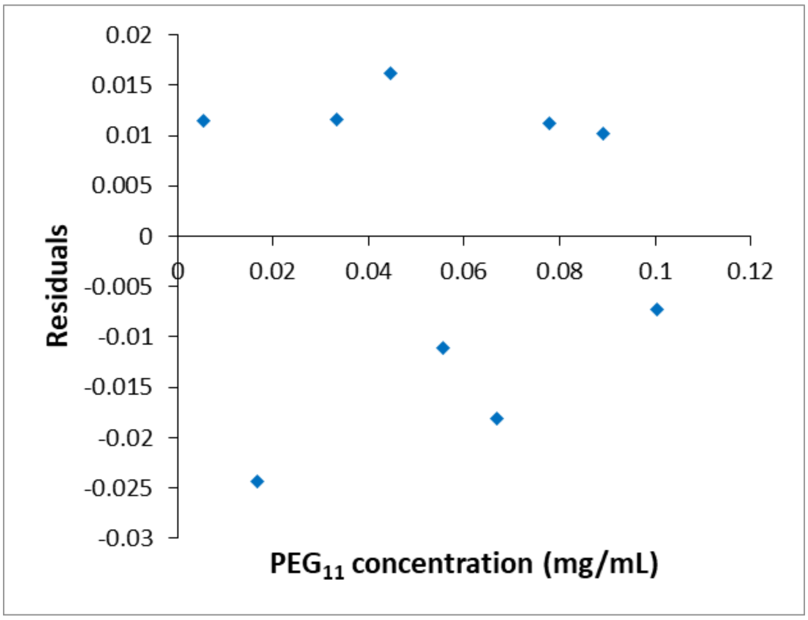

Figure S2. Residual plot of the quadratic regression model of the calibration data for PEG_{11}.
